# Framing a novel holistic energy subsystem structure for water-energy-food nexus based on existing literature (basic concepts)

**DOI:** 10.1038/s41598-023-33385-8

**Published:** 2023-04-18

**Authors:** Masoud Vahabzadeh, Abbas Afshar, Amir Molajou

**Affiliations:** Civil Engineering Department, Iran University of Science & Technology, Tehran, Iran

**Keywords:** Ecology, Environmental sciences, Hydrology, Energy science and technology

## Abstract

It is interesting to note that the country of Iran is essential in terms of energy production and consumption, and the economy of Iran is mainly dependent on energy revenues. Therefore, thermal and hydropower plants consume water to produce various energy carriers. Considering that Iran is suffering from water stress, the nexus of water and energy becomes very important. This paper frames a comprehensive structure for Iran's energy subsystem within the Water, Energy, and Food (WEF) nexus system. The energy subsystem's supply and demand side in the proposed framework are formulated using data and physic-based equations. The presented framework addresses most interactions between WEF subsystems in a dynamic and adaptive setting. It is shown that through analysis of binding interactions between WEF, different management scenarios can boost the flexibility of the supply and demand side of the energy subsystem. In addition, by incorporating this framework, the water subsystem will manage the allocated and consumed water on the supply side and arrive at the most desirable outcome for the water sector. Also, the optimal cropping pattern could be evaluated based on energy consumption.

## Introduction

Global resource demand is growing due to climate change, urbanization, and population growth. Water, Energy, and Food (WEF) demand will rise by 50% by 2050 compared to 2015^1–3^. By recognizing limited resources, the alarming demand increase may strain WEF resources. Extracting fossil fuels like oil, gas, coal, Etc. to provide more energy harms natural resources over time^4^.

It has been reported that many people are suffering from undernutrition. Statistics show that around 784 and 821 million people suffered from hunger in 2014 and 2017, respectively^5,6^. More land has been farmed to meet global food demand. Deforestation and land use changes have led to climate change, which is undesirable^7,8^. Moreover, World Health Organization (WHO) estimates that over 785 million people do not have adequate access to drinking water, and over 884 million lack safe drinking water^9^. An additional 1.4 billion people used essential services, and five billion used safe drinking water. Additionally, more than 206 million people subsisted on limited services, 435 million relied on unimproved sources, and 144 million still used surface water^10^. Water stress will make 700 million of the world's population migrate to a region with enough water, resulting in a war between nations if they cannot enter the area with plenty of water^11^.

Besides, the statistics show that 840 million people worldwide lack access to electricity in rural areas, and three billion people cannot access clean cooking fuels^11^. The temperature will rise by 1 °C compared to the Paris agreement standard^12^. If the melting trend continues, oceans, seas, and lakes will rise. According to this forecast, 150 million people will be under the tide line by 2050, and 360 million will face hazardous phenomena by 2100^13^. The rate of greenhouse gas emissions must be reduced by about 55% compared with 2010 if the planet's temperature is limited to 1.5 °C^7^.

### Previous management approaches

The shocking statistics and reports have led to solutions being proposed. The Food and Agriculture Organization (FAO) developed the "Twin approach" to solve food shortages in the 2000s^14–16^. Various approaches to their comprehensive management were proposed regarding water resources, including Integrated Water Resources Management (IWRM), but none examined their relationship with other resources^17^. One resource has a separate management policy from the other two, resulting in contradictory results. Also, competition is higher because WEF resources are considered their critical interactions. Earlier approaches, such as the Twin approach and IWRM, are now criticized as incomprehensive in the new environment where the population overgrows, lifestyles change, and demand for resources increases dynamically^18^.

### WEF nexus system approach emergence

**Figure 1:**
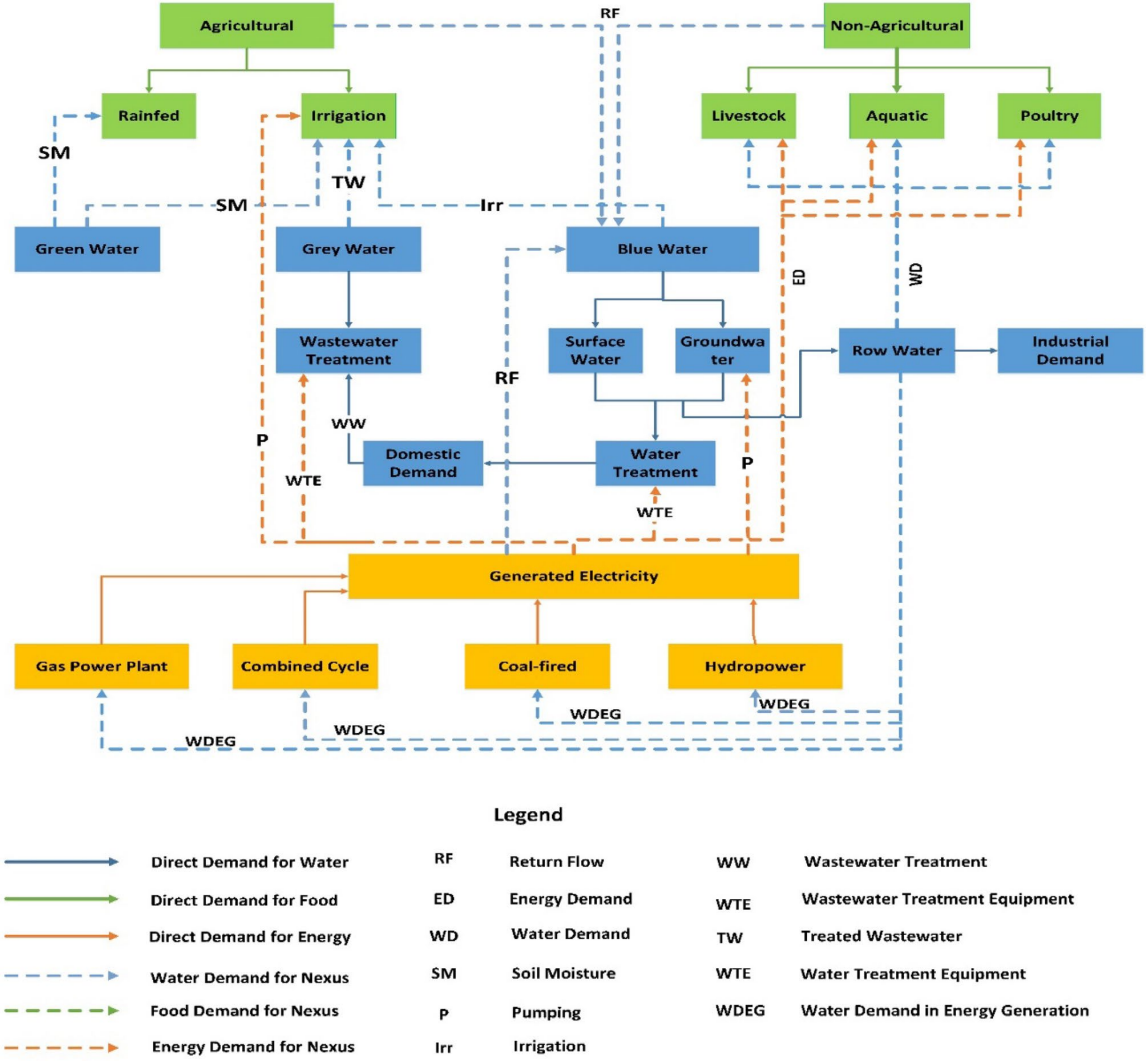
The WEF resources nexus interactions^18,31^.

Several studies have determined that management solutions tailored to WEF resources individually lead to unsustainable use of these resources^19–25^. So, it may have been possible to achieve water security through policies, but food and energy security might have been neglected, and it was the first time that has been mentioned in the world^26^. For instance, in this regard, while Qatar has attempted unsuccessfully to reduce its reliance on food imports by adopting self-sufficiency policies, the water and energy security of the country has been severely affected^27^. Management approaches must undergo a paradigm shift to meet current needs and future sustainable development goals. A multi-centric study of WEF resources can lead to a wealth of knowledge about their relationship and complexity, leading to the emergence of the WEF nexus in recent years^26,28–30^. Since natural resources such as WEF sources are closely linked, studying their interactions is challenging. The interactions among WEF resources nexus are shown in Fig. 1.

### Function and aims of the WEF nexus system approach

In the WEF nexus approach, actions in the water sector led to changes in energy and food subsystems, so the decision-making pattern will be coordinated in WEF subsystems^28,32^. The WEF nexus is tasked with exploring how three WEF subsystems will grow together and interact with one another^33,34^. Furthermore, synergies and tradeoffs will be grown due to their management in the nexus system approach^19,35–37^. Finally, the nexus approach aims to achieve WEF security by considering interdependencies between WEF components^38,39^.

### Energy subsystem importance within the WEF nexus system approach

**Figure 2:**
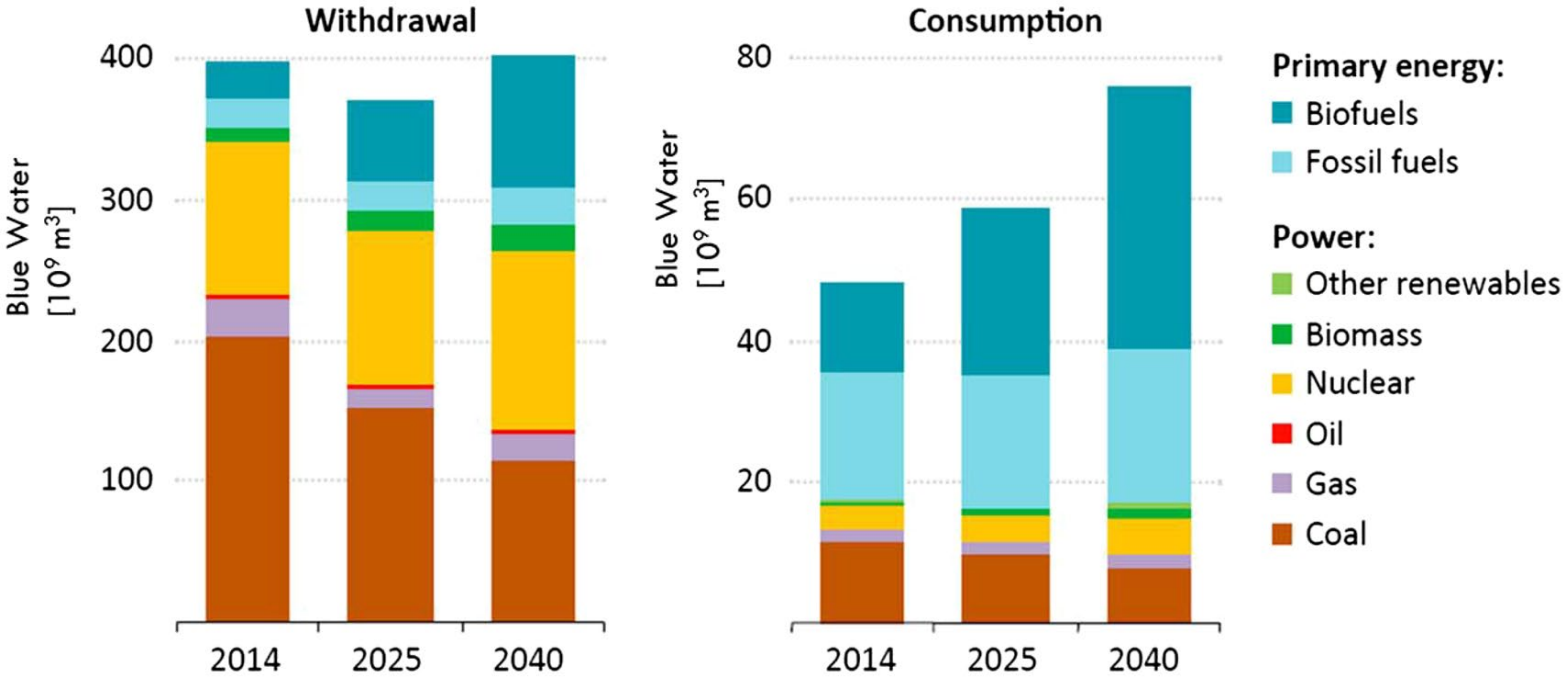
Water consumption and withdrawal for energy production^40^.

The energy subsystem is one of the WEF subsystems that consume considerable water resources. Water resources are significantly affected by thermal and chemical pollution associated with energy production. Water scarcity makes energy vulnerable because it is dependent on water availability. Water and energy are closely related, so ensuring a sustainable supply requires a nexus approach. The energy sector accounted for 3% of total water consumption and 10% of total withdrawals in 2014^40^. Almost 64% of the consumption and 12% of the withdrawals, the water was used to extract energy sources^40^, and the rest was used to generate power (see Fig. 2).

The energy subsystem consumes a lot of water, according to statistics. Therefore, it is necessary to manage the energy subsystem's water consumption using the WEF nexus management approach to see the effects of different actions under different scenarios.

Much energy is used in agriculture. Agricultural activities use energy directly or indirectly. Machines, agricultural equipment, fertilizers, and pesticides are indirect consumers. The food subsystem uses 30% of the world's energy, and 6.6% is used to produce primary products, livestock, and fish. Many mechanized operations exist in modern agriculture. Most of the work is mechanized, and agricultural operations like tillage, planting, irrigation, and harvesting are efficient. All these activities need gas or electricity^41^.

The WEF nexus system lacks a framework for evaluating resource allocation strategies. Modeling the interactions between different sources in the WEF nexus system and examining the relationships between different elements can help managers and decision-makers make accurate and appropriate decisions to integrate the mentioned challenges among diverse stakeholders^18^. Different studies have been conducted to model the relationship between WEF subsystems on regional, national, and local scales, including the WEF Nexus Tool 2.0^22^, (Multi-Scale Integrated Analysis of Societal and Ecosystem Metabolism) MuSIASEM^42^, and (Nexus Simulation System) NexSym^43^.

### Previous WEF nexus system simulation literature focusing on energy subsystem

The WEF nexus models developed before were used in different case studies. Some WEF nexus models evaluate primary component connections^44–46^. The ANEMI model, which utilizes a system dynamics approach, was created as a WEF nexus model to consider the feedback relation between climate, biosphere, and society^47^. The ANEMI model includes water quality, land use, population, water demand, surface water, climate-carbon cycle, and economy modules. The ANEMI model simulates module feedback and answers 'what-if?' questions. ANEMI is not used to simulate each module in detail. In this model, the energy subsystem was not as comprehensive as water, focusing on water rather than the other two subsystems.

To some extent, some problem of the energy subsystem in the ANEMI was resolved. MuSIASEM was developed as an integrated model in that the interconnections among WEF are modeled^42^. It was applied to assess the alternative energy (biofuel production) in Punjab, India, and electricity production in the Republic of South Africa. The WEF nexus is modeled in this framework by evaluating features of the metabolic patterns of WEF. Unlike the ANEMI model, this model managed to consider the supply side of the energy subsystem, but it could not thoroughly consider the supply side of energy.

In 2015, WEF Nexus Tool 2.0 was developed, and the energy subsystem's supply-side and demand-side were considered^22^. In this model, WEF requirements are modeled on a multiscale to achieve food self-sufficiency. With this model, restricted resource feedback evaluation is determined. This model was used in a national case study to evaluate scenarios for sustainable resource management in Qatar. It is crucial to realize and consider the interactions between essential WEF nexus components, but in this model, the water and energy required to produce energy carriers and some primary demand and supply-side components are not stated.

Subsequently, in 2017, the NexSym model was developed^43^. It is a new system dynamics-based model. NexSym uses technological, ecological, and consumption components to assess WEF supply and consumption. This model analyzes supply and consumption holistically but locally. The energy supply side lacks thermal power plants and hydropower due to its small scale, so it cannot be implemented on a large scale.

In 2021, a WEF nexus simulation model was built using a Stock-Flow Diagram (SFD) of WEF Security in a local context to analyze the effects of three proposed policy interventions in Karawang Regency, Indonesia^48^. The SFD and simulation were constructed through the STELLA Professional software, based on a previously established qualitative causal loop model of the same system (the Karawang WEF Security (K-WEFS) model). The supply side of this model included a solar power plant, while thermal power plants and the demand side of the energy subsystem were not modeled.

In 2022, the BJ-FEW model was developed using the STELLA platform, a system dynamics model that incorporated both production and consumption aspects of WEF systems into a single system-of-systems model^49^. This model considered the interactions between WEF sectors within and beyond the urban economic system and was run for Beijing over 2000–2050 to simulate changes in WEF demands and supply. The energy subsystem section of this model omitted the water consumption of thermal power plants and the net head of hydropower as nexus variables to implement interactions; however, the demand side of this subsystem was modeled comprehensively.

Previous models' flaws were resolved by the WEF Nexus Simulation Model (WEFSiM) and WEF-Sask model^50–52^. By analyzing resource interactions, these models could consider supply and demand on a national scale. The energy demand section of the energy subsystem is a database because it is based on resource production intensities. Demand-side components like groundwater pumping should be simulated to consider interaction variables. The energy subsystem needs water subsystem variables to calculate pumping energy demand. The water subsystem determines nexus variables. Nexus variables are exchanged between subsystems to calculate a variable.

### Contribution of study

In most previous WEF nexus models, supply and demand were not modeled adequately and holistically. As a result, the energy subsystem lacks a comprehensive framework that can be applied on a large scale and considers WEF nexus model interactions. Previously, WEF nexus system models did not separate the amount of water withdrawn and consumed by thermal power plants. The amount of water withdrawn and consumed by thermal power plants varies based on their type. Furthermore, the net head of reservoirs and the amount of water flowing through turbines were not considered in the section on hydropower plants. It is impossible to adequately consider the interactions between the energy and other subsystems when the mentioned nexus variables are not considered. In general, the energy subsystem's supply and demand sides have not been accurately modeled and modeled with complete accuracy in WEF nexus system modeling. In the WEF nexus model, energy subsystem frameworks can consider different scenarios and answer 'what-if' questions. This study proposes a holistic framework for Iran's energy subsystem in the context of the WEF nexus system by collecting data and relations from existing literature.

Fragmented energy data for food and water and water data for energy subsystems require a single comprehensive database and equations for Iran's WEF nexus system approach. The literature was used to identify energy- and water-intensive activities within the energy subsystem and starting points for reducing energy and water consumption. By using these data and equations, sustainability analysis tools can assess and secure the environmental performance of the entire energy subsystem, including supply and demand.

Unlike the energy frameworks used within the previous WEF nexus models that were mostly databased, our framework is based on the combination of data and equation-based to formulate the supply and the demand side of the energy subsystem in national and sub-national scales to meet the WEF nexus system's requirements. All the needed statistical data must be gathered to accomplish such a framework, including energy for water, food, energy for energy, and water for energy. Thus, in this paper, we explored works of literature in-depth to create a comprehensive framework for the energy subsystem.

## WEF resources condition in Iran

Managing water and the environment effectively is a pressing issue in Iran^53,54^. As water demands continue to rise due to declining natural water supply and newly developed surface water and groundwater resources, the country's technological approach addresses water shortages through an extensive network of dams, inter-basin water transfer projects, and groundwater withdrawal has proven inadequate^55–57^. As a result, Iran is struggling with a state of "water bankruptcy" that endangers the future of one of the world's oldest and most prosperous civilizations^55^. The country's water management problems will likely worsen as water stress rises. Extensive drying up of water bodies, frequent sand and dust storms, widespread groundwater table decline, deteriorating water quality, and increasing competition and conflict over limited water resources all point to water security becoming a primary concern from a national security standpoint^53^. Prolonged droughts have contributed to the political unrest and social instability that have plagued countries like Syria in the Middle East and North Africa (MENA) region^58^.

**Figure 3:**
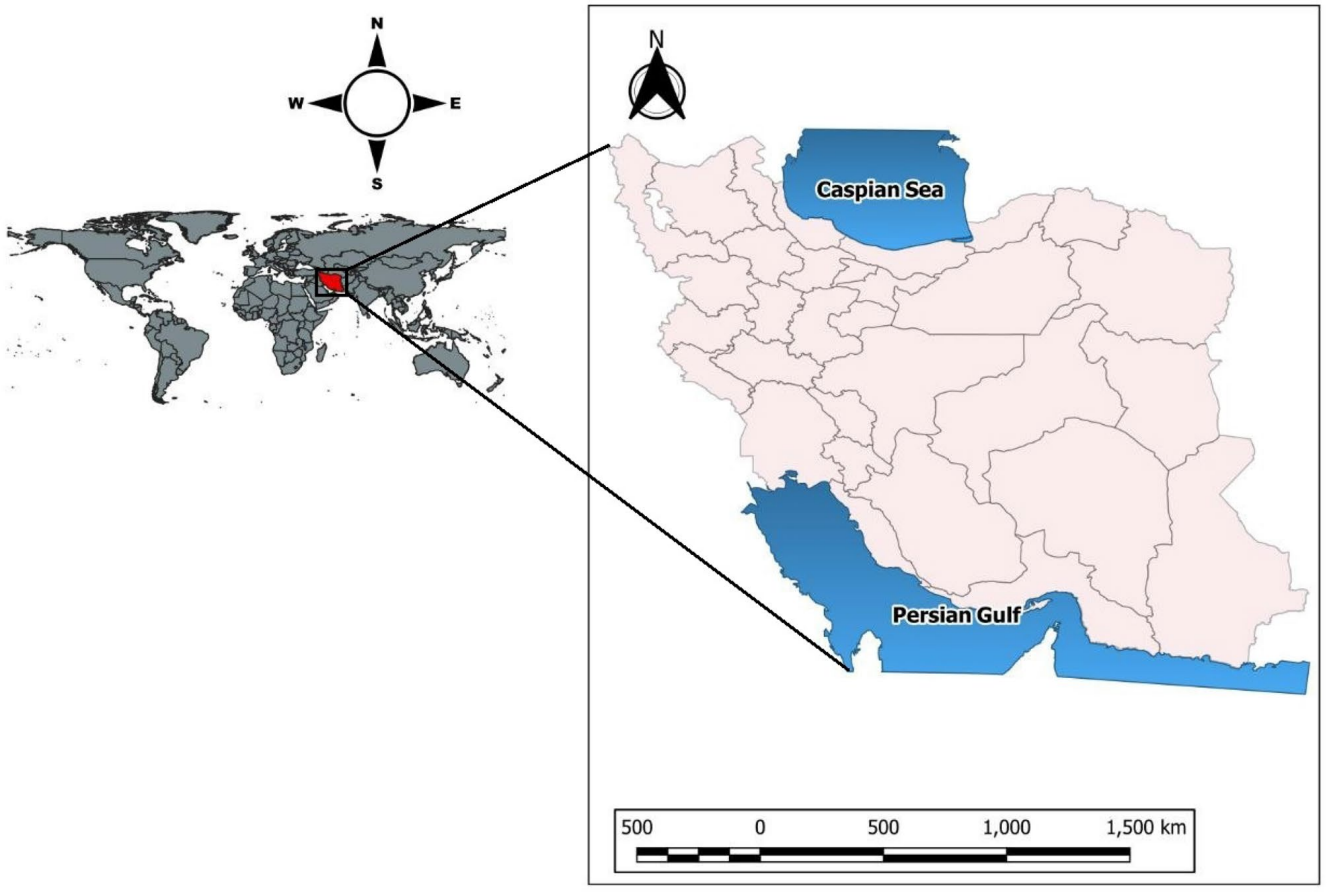
Iran country map.

There is little to no cost for access to water in Iran because the government regulates it. As a result, Iran's agricultural expansion policies have relied heavily on easy access to low-cost energy and water (both surface and groundwater). Agriculture accounts for roughly 10% of GDP and employs around 20% of the population^53^. This sector also consumes over 90% of Iran's total water withdrawals^53^. Due to severe water shortages, as evidenced by low surface water levels and a significant drop in the groundwater table, agricultural activities are effectively limited rather than prevented by prohibitive water and energy prices. Variable water scarcity can be attributed to the country's highly variable climate, which ranges from arid and semi-arid in most regions to subtropical in the narrow strip of land that borders the Caspian Sea (see Fig. 3). Iran receives about a third as much precipitation as the rest of the world on average. The average annual rainfall is less than 100 mm but can exceed 1000 mm in remote areas^53^. As a result, irrigated agriculture in the country depends on groundwater due to the unreliability of the surface water supply.

**Figure 4:**
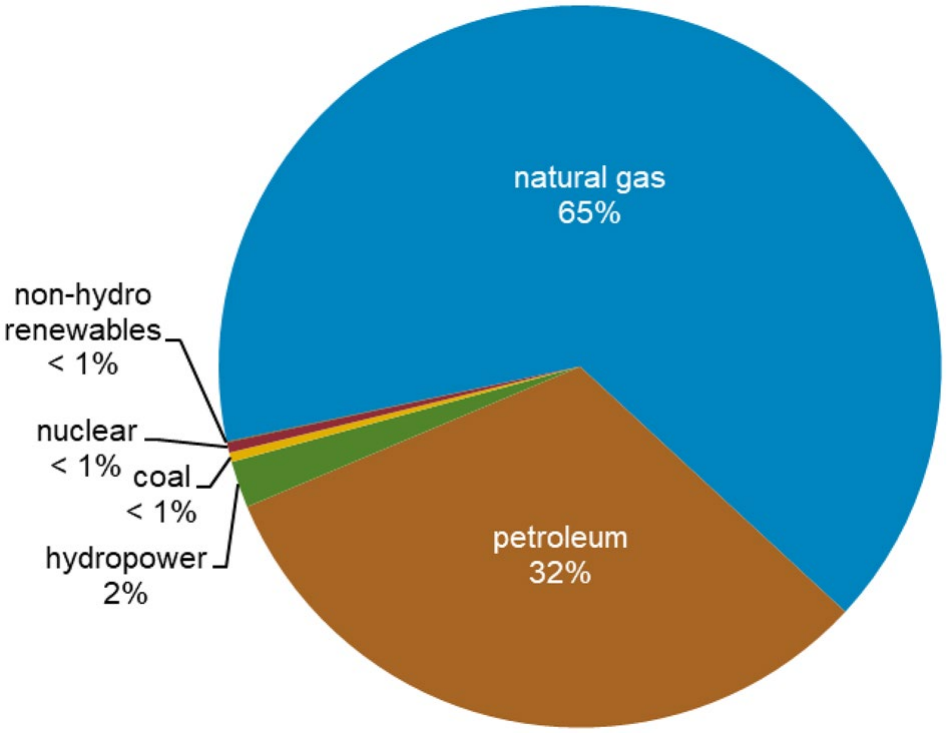
Iran's total primary energy consumption, share by fuel, 2019^61^.

In addition, although petroleum and other liquid revenues are essential to Iran's economy, the country is more diversified than many others in the Middle East. According to the International Monetary Fund, in the most recent fiscal year for which data is available (April 2016–March 2017), revenue from crude oil exports constituted nearly 40% of Iran's total government revenues^59^. In 2019, Iran was the largest energy consumer in the Middle East, consuming an estimated 11.7 quadrillion British thermal units of primary energy^60^. Natural gas and oil constituted nearly all of Iran's total primary energy consumption, with marginal contributions from hydropower, coal, nuclear, and non-hydropower renewables (see Fig. 4).

**Figure 5:**
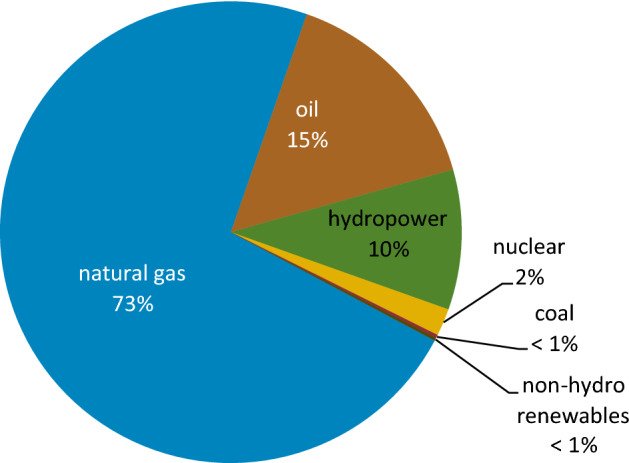
Iran's electricity generation capacity by fuel, 2019^61^.

Iran produced 306 terawatt hours (TWh) of net electricity in 2019, with 88% of that amount originating from fossil fuel sources^62^. Iran's largest source of fuel for electricity generation is natural gas, which accounts for nearly 73% of total generation. In 2019, 15% of Iran's electricity production was fueled by oil, up from 9% in 2018^61^. The remaining fuel sources used to generate electricity in Iran are coal, hydropower, nuclear, and non-hydropower renewables (see Fig. 5). Due to heavy, widespread rainfall and flooding, Iran's hydroelectric power output doubled from nearly 16 TWh in 2018 to 30 TWh in 2019, the highest increase in the generation on record^61^. In 2019, hydropower accounted for 10% of Iran's total generation, displacing some oil- and natural gas-generated electricity^61^.

It should be noted that agriculture and energy production are critical to Iran's economy. Water is consumed to produce energy, such as the extraction of fossil fuels and the generation of energy from thermal and electric power plants. However, due to the poor condition of Iran's water resources, the country's economic situation may change due to its reliance on energy production. In the circumstance of a water crisis caused by poor water resource management, thermal and electric power plants may be unable to produce energy, and fossil fuel extraction and exports may be hampered.

On the other hand, agriculture is a major economic component of Iran, and it heavily relies on energy to produce crops. In the circumstance of a lack of water resources and, as a result, a decrease in energy production, agricultural production will also decrease. Until now, Iran has lacked a comprehensive and appropriate framework for the energy subsystem based on the nexus approach. Based on existing literature, this research was able to provide a suitable framework for energy for the country of Iran.

## Methodology and the proposed framework

The existing comprehensive and stand-alone energy models, such as LEAP^63^ and OSeMOSYS^64^, may be expanded to fit into a WFE nexus system. However, such modification for all WEF subsystem interactions in an online template is complex and computationally expensive. Therefore, this new framework for the energy subsystem was developed to meet the WEF nexus system requirements within the online information exchange template^65,66^. With a focus on nexus modeling, the proposed energy framework accounts for demand-side and supply-side measures with online and direct data and information exchange during any simulation step. The proposed framework classifies data and information into four groups with unique characteristics, availability, and adaptability during nexus system simulation. The proposed framework combines statistical data and equations to address demand and supply's spatiotemporal variation. Applying this framework to WEF nexus models allows for sound decision-making. In addition, this framework considers binding WEF interactions, and supply and demand are given more flexibility using different management scenarios.

**Figure 6:**
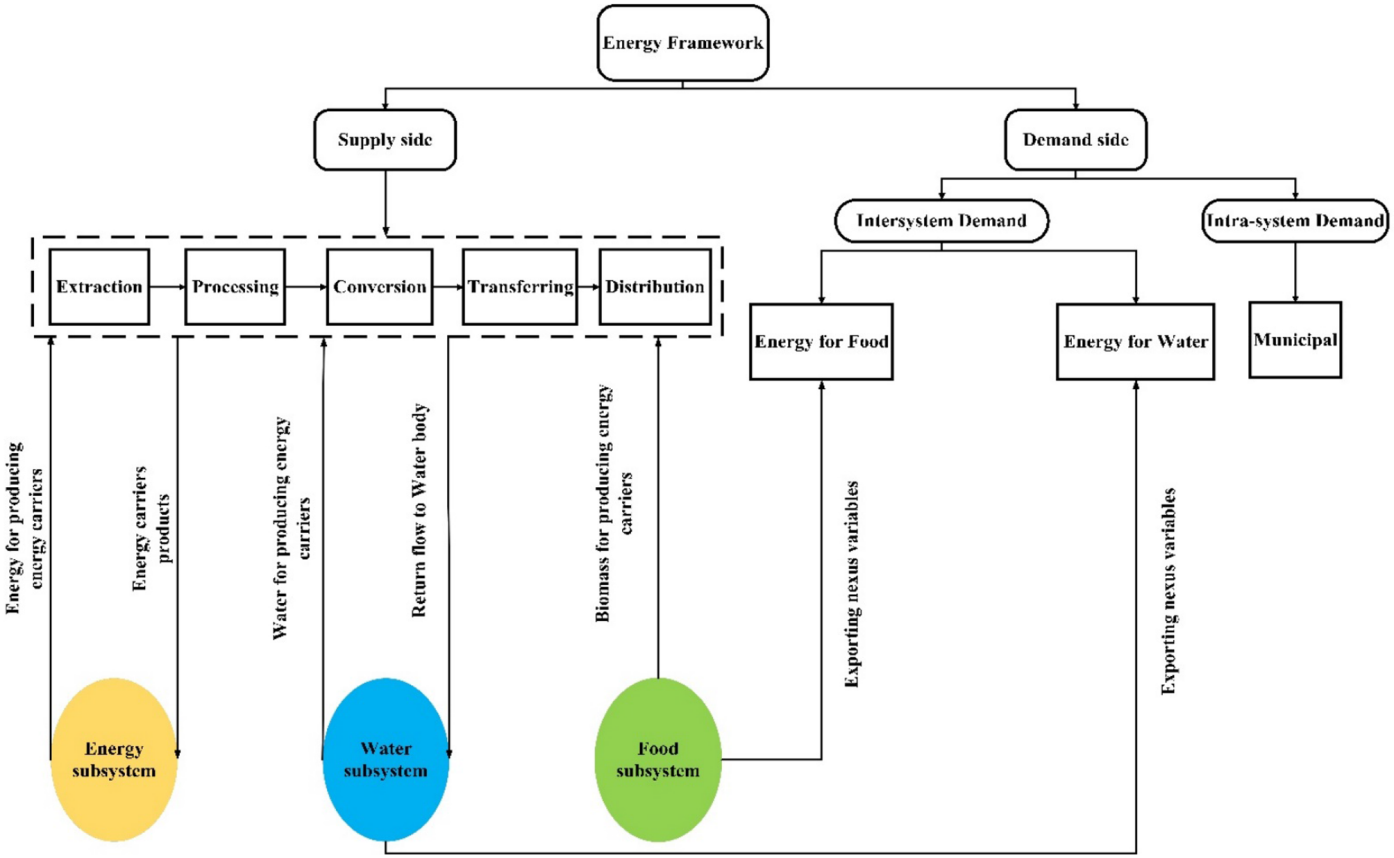
The proposed structure of the energy subsystem.

The novel energy subsystem's framework is categorized into supply and demand sections. The proposed structure of the supply and demand side of the energy subsystem in the context of the WEF nexus system is illustrated in Fig. 6.

As shown in Fig. 6, the energy subsystem in the nexus approach is divided into the supply and demand sides. Both the supply and demand sides interact with WEF subsystems, and a literature review was conducted based on WEF nexus system requirements to collect related equations and database relations. Notably, the energy subsystem's significant interaction on the supply side is with the water subsystem. The proposed framework is described in detail in the following.

### WEF nexus system interactions

#### Energy and water interactions

Water is used to generate energy carriers such as electricity, and petroleum products and to extract fossil fuels. Most of the water consumed in the energy subsystem is related to thermal power plants, which are used in the cooling sector. Without consumed water, producing the aforementioned energy carriers is impossible. Therefore, there is an interaction between energy and water, and the lack of investigation of which leads to the neglect of water management. Therefore, the amount of water allocated to the power plants as well as the net head of the reservoirs are considered nexus variables in the energy subsystem. The interactions of the energy framework on the supply side with the nexus approach are shown in Table 1.

**Table 1:** Interactions among energy and water on supply side with nexus approach.

	Energy	Interaction relationship	Water	Interaction relationship
Energy	Required energy per unit of fossil fuels extraction	Energy intensity data	Amount of firm energy generation	Determining by the decision maker
			Required energy for groundwater pumping	*E* = $\frac{9.8 \left(\frac{m}{{s}^{2}}\right) \times lift \left(m\right) \times mass (Kg)}{3.6\times {10}^{6}\times efficiency (\%)}$
			Required energy for treatment plant	Required energy per unit of water treatment
Water	Required water withdrawal and consumption for thermal and hydropower plants	(1) Water intensity data		
	Required water per unit of fossil fuels extraction	(2) $I$ = $A$ (${HR}$—$B$) + $C$		
		(3) E = Ɣ.Q.h_net_.**ξ**		
		Water intensity data		

I, water consumption intensity; A, amount of water required to dissipate one kilojoule of heat based on the type of cooling system; HR, heat rate input to the power plant; B, total heat outputs from the power plant except for the heat flow dissipated by the cooling system; C, required water in other parts of the power plant except the cooling system; E, generated power; Ɣ, specific weight of water; Q, discharge which comes into the turbine; h_net_, net head of water**; ξ**, efficiency of the plant.

#### Energy and food interactions

Energy is widely used in the agricultural industry. So that energy is consumed directly or indirectly in agricultural lands. Indirect consumption includes the production process of machinery, agricultural land equipment, fertilizers, and pesticides. Also, direct energy consumption in agricultural operations such as tillage, planting, irrigation, and harvesting is highly efficient using agricultural equipment. As a result, all these activities require energy carriers. Therefore, the amount of cultivated area and consumed agriculturalinputs are considered nexus variables for the energy subsystem. On the demand side, the interactions of the energy subsystem within the context of the WEF nexus system are shown in Table 2.

**Table 2:** Interactions among energy and food on the demand side with nexus approach.

	Energy	Interaction relationship	Food	Interaction relationship
Energy			Required energy for agricultural machinery	(1) $ME=E\times \frac{G}{T}\times {Q}_{h}$
			Required energy for agricultural inputs production	(2) Energy intensity data
			Required energy for labor	(3 )${E}_{l}={W}_{l}\times {E}_{i}$
Food	Required biomass per unit of energy generation	Biomass intensity data		

ME, machinery energy; E, production energy of the machine; G, weight of the machine; T, economic life of the machine; Q_h_, total working hours of the machine in a season; E_l_, human labor energy; W_l_, number of workers per hectare; E_i_, energy use per worker.

It is worth mentioning that the tradeoff exist among WEF subsystems. For example, Integrating renewable energy sources into the supply sector is essential to the WEF nexus system. Solar and wind power plants are considered alternative energy sources that play a vital role in the energy subsystem. In addition, climate change presents a challenge in the form of increased evapotranspiration in crops, leading to an increase in irrigation needs that forces a tradeoff between energy and food subsystems for water withdrawal. In comparison, renewable energy sources require less water than conventional sources, significantly reducing interaction between the energy and water systems and reducing the tradeoff between agriculture and energy production^65–67^.

### Supply-side

**Figure 7:**
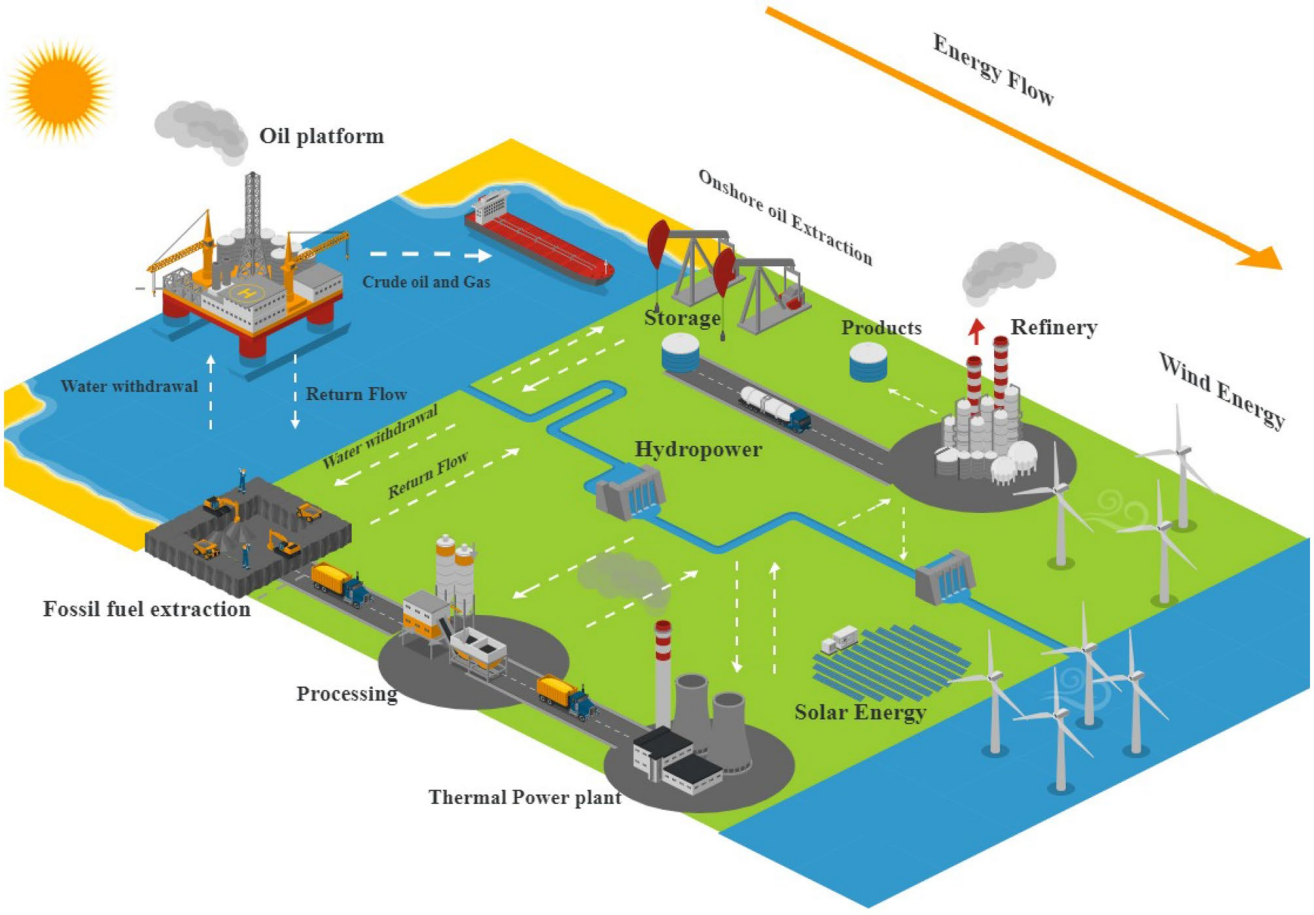
The structure of the Supply-side of the energy subsystem in the context of the WEF nexus system.

The energy supply side has many subsets. The supply side of the energy subsystem includes the flow of primary and secondary energy. The energy supply side consists of technologies for processing, converting, transmitting, and distributing energy carriers to meetsocietal demand. Natural resources provide energy's raw material as fuel, which must be processed and converted into primary energy. Figure 7 depicts the energy supply side of the WEF nexus system.

**Figure 8:**
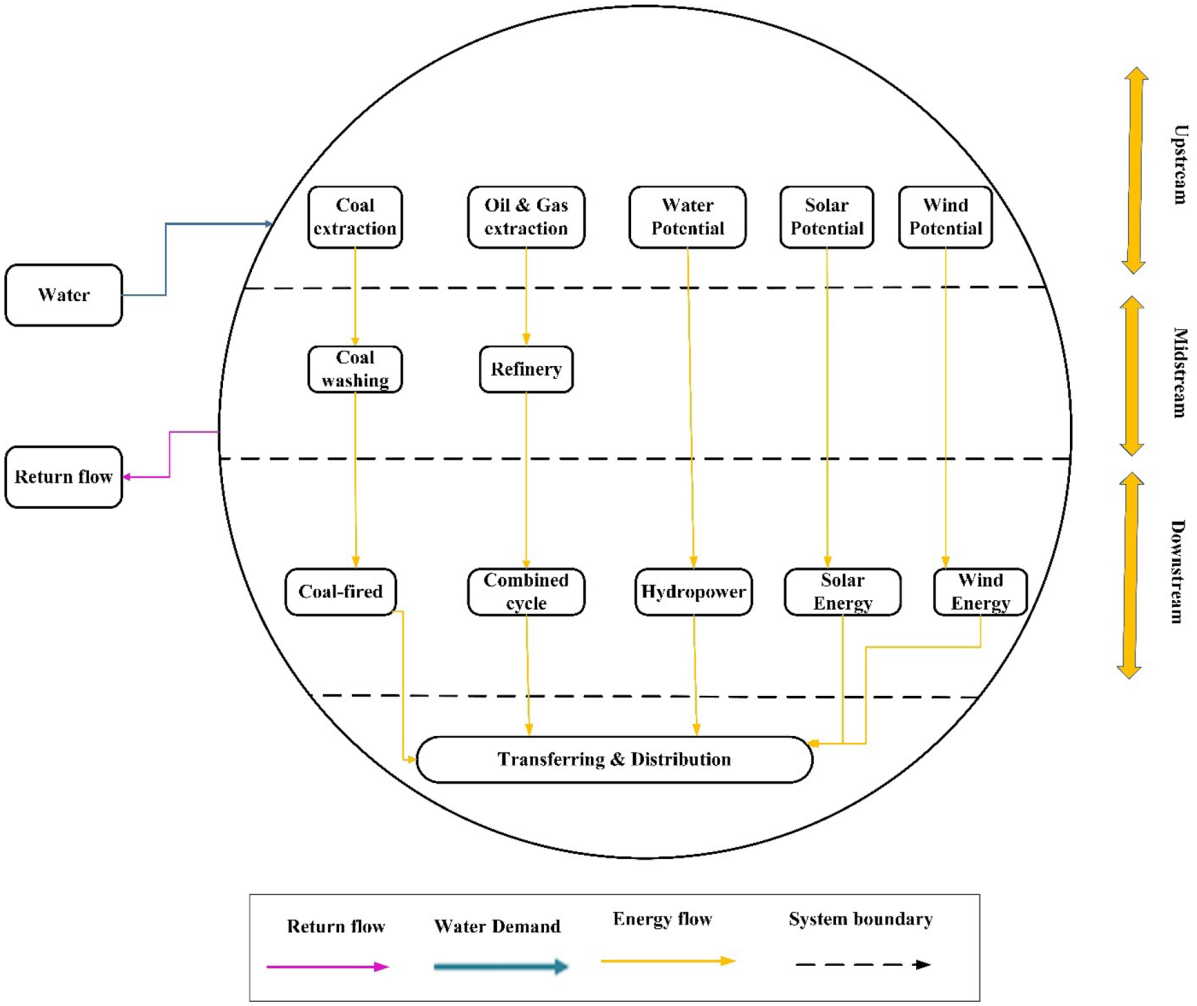
Supply-side of energy subsystem's elements.

Most of the necessary WEF nexus interactions have been considered on the supply side of the energy subsystem structure. For instance, oil fields require water and energy to extract crude oil, and refinery units and thermal power plants require much water for tower cooling. To model the supply side of the energy subsystem in the WEF nexus system, the thermal power plants, refineries, Etc., must be included. So, this large and complicated system should be diagrammed as energy flow. Figure 8 depicts the upstream, midstream, and downstream supply-side energy flow.

#### Upstream flow of energy

Upstream flow is related to extracting fossil fuels. Non-renewable fuels require water and energy to extract, but solar, wind, Etc. do not. Therefore, water and energy consumption and returned water at the upstream border should be examined to extract non-renewable fuels. Databased relations can provide water and energy consumption data for each unprocessed fuel unit. At the upstream flow boundary, unprocessed fuel is extracted, and water is returned.

#### Midstream flow of energy

Midstream flow is related to the extracted crude fuel processing. The processing process also requires water and energy. Data on water and energy consumption in the processing units are obtained through database relations. The output of the midstream flow is the amount of processed fuel and the return water.

#### Downstream flow of energy

Downstream flow is related to converting different types of primary energy carriers into different secondary energy carriers and the transfer and distribution of energy carriers. Most of the water consumption in the downstream sector is related to cooling systems in power plants, and in practice, water and energy consumption of transmission and distribution technology sectors is negligible. The pertinent data about water consumption and energy carrier generation is statistical.

#### Two important terminologies on the supply-side

Withdrawal and consumption are essential supply-side of the energy subsystem terms. Water withdrawal is water extracted from water bodies for a specific purpose, and water consumption is the water reduced from the extracted water and wholly consumed in the process^68^. It should be noted that the return flow is the subtraction of water withdrawal and water consumption.

The energy subsystem's many parts make it a complex system. The structure of the supply side of the energy subsystem in the WEF nexus system has increased its complexity by considering water withdrawal, water consumption, and energy consumption. To simplify supply-side modeling, part of it is formulated by simple equations, and the other part is based on data. The framework of the energy subsystem is formulated at two spatial scales, national and subnational. Considering these spatial scales is crucial because we can benefit from planning at national and sub-national (basin) levels within the WEF nexus system.

#### Upstream flow formulation

The formulation of the upstream flow boundary is databased, meaning the intensity of water and energy consumption is considered instead of the equation. Noticeably, municipal demand is not considered within this framework. Instead, WEF nexus demands are highlighted, including energy for water and food. Thermal power plants and refineries are expected to use fossil fuels, so the energy requirements for electricity generation, diesel, gasoline, Etc., will be taken into account from upstream to downstream all at once (see Table 3). The water withdrawal, water consumption, and energy consumption data are illustrated in Tables 3 and 4. It should be noted that the formulation is considered the same for national and sub-national scales due to the complexity of upstream flow.

**Table 3:** Water withdrawal and consumption data per unit of energy.

Energy category	Sub-category	Water consumption (m^3^/MWh)			Water withdrawal (m^3^/MWh)			References
		Median	Min	Max	Median	Min	Max	
Coal	Surface mining	0.083	0.023	0.22	0.083	0.023	0.23	^69^
	Underground mining	0.211	0.064	0.87	0.216	0.064	0.87	^69^
Gas	Conventional	0.015	0.004	0.098	0.019	0.015	0.129	^69^
	Unconventional	0.061	0.011	0.79	0.064	0.019	0.83	^69^
Oil	Conventional	0.07–1.96	0.07–1.96	0.07–1.96	–	–	–	^70^
	Unconventional	0.43–4.21	0.43–4.21	0.43–4.21	–	–	–	^70^

**Table 4:** Energy intensity data for fossil fuels extraction in the upstream flow.

Energy type	Unit	Amount	References
Coal	MJ_h_/MJ_e_	2.6	^70^
Lignite	MJ_h_/MJ_e_	2.92	^70^
Natural gas	MJ_h_/MJ_e_	1.96	^70^
Biomass	MJ_h_/MJ_e_	2.5–5.0	^70^
Oil	MJ_h_/MJ_e_	2.58	^70^

Transportation is also required to move the extracted fossil fuels to the midstream flow. It should be noted that the transportation system boundary is associated with the upstream flow. The water consumption data for the transportation section is shown in Table 5.

**Table 5:** Water intensity data for fossil fuels transportation in the upstream flow.

Energy category	Transportation type	Water withdrawal (m^3^/MWh)			Water consumption (m^3^/MWh)			References
		Min	Median	Max	Min	Median	Max	
Coal	Railway	0.0011	0.0038	0.0076	0.0003	0.0011	0.0038	^69^
	Slurry pipeline	0.38	0.42	1.55	0.38	0.42	1.55	^69^
Gas	Pipeline	0.0011	0.0022	0.0034	0.0003	0.0009	0.0016	^69^
	Transport (liquefied natural gas)	0.032	0.032	0.032	0.004	0.004	0.004	^69^

Tables 3 and 5 show that water withdrawal and consumption may be the same in some cases. Water withdrawal is equal to consumption in these specific instances since data are only available for one of the two cases. In Tables 3 and 5, the amount of water allocated to the extraction of fossil fuels and the transfer of these fuels to the processing units is considered a nexus variable for the energy subsystem.

#### Midstream flow formulation

The fuels obtained from this stage are processed fuels. The formulation of this stage for the sub-national (basin) scale is based on Eqs. (1–6) as follows^71^:

1 $$E_{u,t}^{OR} = F_{u,t} . \eta_{u,t}^{OR} \;\;\forall_{t = 1, \ldots ,T} , \forall_{u = 1, \ldots U}$$

2 $$E_{u,t}^{GR} = F_{u,t} . \eta_{u,t}^{GR} \;\;\forall_{t = 1, \ldots } , \forall_{u = 1, \ldots U}$$

3 $$Cap_{u,t}^{OR} \ge E_{u,t}^{OR} \;\;\forall_{t = 1, \ldots ,T} , \forall_{u = 1, \ldots U}$$

4 $$Cap_{u,t}^{GR} \ge E_{u,t}^{GR} \;\;\forall_{t = 1, \ldots ,T} , \forall_{u = 1, \ldots U}$$

5 $$Cap_{u,t}^{OR} \le Cap_{u}^{MAX - OR} . y_{u} \;\;\forall_{t = 1, \ldots ,T} , \forall_{u = 1, \ldots U}$$

6 $$Cap_{u,t}^{GR} \le Cap_{u}^{MAX - GR} . y_{u} \;\;\forall_{t = 1, \ldots ,T} , \forall_{u = 1, \ldots U}$$

${E}_{u,t}^{OR}$ and ${E}_{u,t}^{GR}$ represent the oil refineries and gas refinery's products such as gasoline, diesel, Liquefied Petroleum Gas (LPG), and Compressed Natural Gas (CNG) in the unit *u* and time step *t* $. {Cap}_{u,t}^{OR}$ and ${Cap}_{u,t}^{GR}$ are capacity of oil and gas refineries, ${\eta }_{u,t}^{OR}$ and ${\eta }_{u,t}^{GR}$ indicate the thermal efficiency. Also, ${Cap}_{u}^{MAX-OR}$ and ${Cap}_{u}^{MAX-GR}$ are maximum capacity of oil and gas refineries, and the binary variables ${y}_{u}$ indicate the existence of the refinery unit. If ${y}_{u}=0$ the refinery unit exists, and if ${y}_{u}=1$, then the refinery unit does not exist. The constraint of fossil fuel production has not been considered in this study.

Furthermore, on the national scale, due to the increasing spatial scale, the formulation of midstream flow is according to the collected database from the literature. Based on Table 6, fossil fuels and biofuels use different amounts of water for production.

**Table 6:** Water intensity data in the midstream flow.

Energy type	Processing	Water withdrawal (m^3^/MWh)			Water consumption (m^3^/MWh)			References
		Min	Median	Max	Min	Median	Max	
Coal	Coal washing	0.034	0.068	3.79	0.034	0.068	3.79	^69^
Oil	Refinery	0.00728	0.0112	0.01344	0.00728	0.0112	0.01344	^72^
Gas	Refinery	2.63E−05	0.000078	0.000132	2.63E−05	7.89E−05	0.000132	^69^
Bioethanol	Processing	0.02576	0.0406	0.0812	0.02576	0.0406	0.0812	^72^
Biodiesel	Processing	0.00868	0.00868	0.00868	0.00868	0.00868	0.00868	^72^

According to Table 6, the amount of water allocated to the processing units in the midstream is considered a nexus variable for the energy subsystem.

#### Downstream flow formulation

The downstream flow of the supply side of the energy subsystem is associated with electricity and steam generation. At this stage, the power plants are divided into (1) thermal power plants and (2) renewable power plants. For instance, coal-fired and combined cycle power plants are thermal power plants, while hydropower and wind power are renewable.

#### Thermal power plants formulation on the national and sub-national scale

Thermal power plants generate electricity using coal-fired, combined cycle power plants, Etc. Notably, vast amounts of water during this stage are used in cooling systems applicable to thermal power plants and hydropower. The related formulation of these mentioned power plants on the sub-national (basin) scale in the operational form is according to Eqs. (7) and (8)^73,74^:

7 $$I = A\left( {HR_{{}} - B} \right) + C$$

where $I$ (Lit/KWh), $A$ (Lit/KJ), ${HR}$ (KJ/KWh), $B$ (KJ/KWh) and $C$ (Lit/KWh) are water consumption intensity, the amount of water required to dissipate one kilojoule of heat based on the type of cooling system, heat rate input to the power plant, the total heat outputs from the power plant except for the heat flow dissipated by the cooling system, and required water in other parts of the power plant except the cooling system. Accordingly, the amount of water needed by the power plant ($I$) depends on the amount of heat dissipated by the cooling system (${HR}$*-* $B$), the type of cooling system ($A$), and other water requirements of the plant ($C$). There is an inverse relationship between the efficiency and the heat rate of the power plant, and the efficiency of each power plant is assumed to be a particular value during the simulation period. Equation (8) is used to calculate the heat rate^73,74^:

8 $$Efficiency=\frac{3600}{HR}$$

Related thermal power plant water consumption and cooling system parameters for Iranian thermal power plants are shown in Tables 7, 8.

**Table 7:** Thermal power plants' parameters value^74^.

Plant type	HR (KJ/KWh)	B (KJ/KWh)	C (Lit/KWh)
Gas-fired	7200	5160–5230	0.02–0.03
Steam	9000–9500	5500–5800	0.1–0.2

**Table 8:** Thermal power plants' cooling system parameters values^74^.

Plant type	A_output_ (Lit/KJ)	A_consumptive_ (Lit/KJ)
Once-through	0.022–0.034	0.00022–0.00068
Wet tower cooling	0.00034–0.00077	0.00033–0.00050
Dry tower cooling	0	0

Furthermore, the thermal power plant formulations on the national scale are databased. This is because it will be time-consuming if the relations pertinent to the basin scale are to be utilized nationally. Hence, the relations for the national scale are based on statistical data used in the literature estimates. Tables 9, 10, 11 and 12 show the estimated data for water withdrawal and consumption for types of thermal power plants.

**Table 9:** Coal-fired power plant's water withdrawal and consumption data^69^.

Sub-category	Water consumption (m^3^/MWh)			Water withdrawal (m^3^/MWh)		
	Median	Min	Max	Median	Min	Max
PC: cooling tower	2	0.757	4.92	2.5	1.74	4.54
PC: open loop cooling	0.53	0.27	1.32	132.5	56.78	215.75
PC: pond cooling	2.8	1.13	3.79	37.85	1.14	98.4
PC + CCS: cooling tower	3.56	3.41	3.56	4.92	4.54	5.30
SC: cooling tower	1.89	1.74	2.23	2.27	2.20	2.54
SC: open loop cooling	0.38	0.24	0.45	87.05	87.05	87.05
SC: pond cooling	0.16	0.015	0.24	56.78	56.78	56.78
SC + CCS: cooling tower	3.33	3.22	3.44	4.16	4.16	4.16
CFB: cooling tower	2.12	2.12	2.12	3.79	3.79	3.79
CFB: open loop cooling	0.79	0.79	0.79	75.70	75.70	75.70
IGCC: cooling tower	1.21	0.13	1.67	1.48	0.61	25.36
IGCC + CCS: cooling tower	2.08	1.97	2.27	2.42	1.82	2.80

PC = pulverized coal, sub-critical; SC = pulverized coal, super-critical; CFB = circulated fluidized bed; IGCC = integrated gasification combined cycle; CCS = carbon capture and sequestration.

**Table 10:** Gas-fired power plant's water withdrawal and consumption data^69^.

Sub-category	Water consumption (m^3^/MWh)			Water withdrawal (m^3^/MWh)		
	Median	Min	Max	Median	Min	Max
CC^a^: cooling tower	0.79	0.18	1.14	0.95	0.57	2.88
CC: dry cooling	0.015	0.015	0.45	0.015	0.004	0.015
CC: open loop cooling	0.38	0.076	0.87	34.06	27.25	79.49
CC: pond cooling	0.91	0.91	0.91	22.71	22.71	22.71
CC + CCS: cooling tower	1.44	1.44	1.44	1.93	1.85	1.93
CT^b^	0.19	0.19	1.29	1.63	1.63	1.63
Steam: cooling tower	2.76	2.12	4.16	4.54	4.54	4.54
Steam: open loop cooling	1.10	0.72	1.55	136.26	132.48	140.05
Steam: pond cooling	1.02	1.02	1.02	1.02	1.02	1.02

^a^Combined cycle; ^b^combustion turbine.

**Table 11:** CSP-generated power plant's water withdrawal and consumption data^69^.

Sub-category	Water consumption (m^3^/MWh)			Water withdrawal (m^3^/MWh)		
	Median	Min	Max	Median	Min	Max
Dish Stirling^a^	0.019	0.019	0.019	0.019	0.019	0.019
Fresnel^a^	3.79	3.79	3.79	3.79	3.79	3.79
Power tower: cooling tower	3.07	2.8	3.26	2.8	2.8	2.8
Power tower: dry cooling^a^	0.098	0.098	0.098	0.098	0.098	0.098
Power tower: hybrid cooling^a^	0.64	0.34	0.95	0.64	0.34	0.95
Trough: cooling tower	3.37	2.12	7.19	3.63	3.29	4.16
Trough: dry cooling	0.30	0.12	0.53	0.30	0.12	0.30
Trough: hybrid cooling	1.27	0.42	1.32	1.27	1.27	1.27

^a^Reflecting data limitations and the nature of water use, we assume withdrawal and consumption are equal for all estimates in this category.

**Table 12:** Geothermal-generated power plant's water withdrawal and consumption data^69^.

Sub-category	Water consumption (m^3^/MWh)			Water withdrawal (m^3^/MWh)		
	Median	Min	Max	Median	Min	Max
Binary: hybrid cooling^a^	1.74	0.83	2.65	1.74	0.83	2.65
Binary: dry cooling^a^	1.10	1.02	2.38	1.10	1.02	2.38
Flash	0.04	0.019	1.36	0.068	0.04	0.094
EGS: dry cooling^a^	1.93	1.10	2.73	1.93	1.10	2.73

^a^Reflecting data limitations and the nature of water use, we assume withdrawal and consumption are equal for all estimates in this category.

After calculating the intensity of water consumption of thermal power plants using Eqs. (7) and (8), the amount of water allocated to power plants is known as the nexus variable.

#### Hydropower formulation on the basin and national scale

Hydropower plants are divided into two classifications which include Reservoirs-based and Run-of-River based hydropower. The hydropower, reservoir-based (dam), and Run-off- River on the basin scale is formulated as Eqs. (9) and (10)^75^:

9 $${\text{E}}_{u,t} = {\uprho }.{\text{g}}.q_{u,t} .h_{net,u,t} .{\upxi }_{u} \;\;\;\forall_{u = 1, \ldots ,U} ,\;\;\forall_{t = 1, \ldots ,T}$$

10 $$h_{net,u,t} = h_{w,u,t} - h_{tail,u,t} \;\;\forall_{u = 1, \ldots ,U} ,\;\;\forall_{t = 1, \ldots ,T}$$

where ${\mathrm{E}}_{u,t}$ (N m = J) is the generated power in unit u and time step t, ρ (Kg/m^3^) water density, g (m/s^2^) gravitational acceleration, ${q}_{u,t}$ (million cubic meters) discharge which comes into the turbine, ${h}_{net,u,t}$ (m) net head of water, ${h}_{w,u,t}$ (m) forebay elevation, ${h}_{tail,u,t}$ (m) tailwater elevation, and ${\upxi }_{u}$ is the efficiency of the plant. If the generated energy (${\mathrm{E}}_{u,t}$) to be calculated in one hour, the unit of it will be Wh (Watt-hour). The installed capacity of the plant's unit is determined by turbine performance.

Hydropower plants on the national scale cannot be formulated similarly to the sub-national scale because operational equations are unreasonable. Therefore, the formulation should be databased. Since the energy generation of different hydropower plants in Iran varies, the essential hydropower plants' water withdrawal is shown in Table 13.

**Table 13:** Water withdrawal of Iranian hydropower plants^76^.

Hydropower plant name	Water withdrawal (m^3^/MWh)
Aras	258.55
Mahabad	190
Zayanderud	47
Dez	8.3
Karun-1	7.7
Maroon	49.25
Masjed Soleiman	1.46
Karun-3	9.5
Karun-4	44
Karkheh	244.32
Amirkabir	5.26
Latyan	10.32
Mollasadra	37.21
Doroodzan	802.35
Jiroft	332.59
Sefidrud	398.41
Shahid Rajaee	32.16

For electric power plants, the amount of water passing through the turbines and the net head of the reservoirs are considered nexus variables in the energy subsystem.

#### Solar photovoltaic system model formulation

The energy is obtained from a photovoltaic module. The cause of energy generation from PV modules is the solar radiation and the ambient temperature^77–82^ and it is expressed as:

11 $${E}_{u,t}^{PV}={n}_{u}^{PV}{P}_{u}^{PV}{\eta }_{u}^{PV}{\eta }_{u}^{INV}{\eta }_{u}^{Wire}\frac{{I}_{u,t}^{rad}}{{I}_{u}^{nom}}(1-{\beta }_{u}^{T}({T}_{u,t}^{C}-{T}_{u}^{Cnom}))$$

where ${n}_{u,t}^{PV}$, ${P}_{u}^{PV}$, ${\eta }_{u}^{PV}$, ${\eta }_{u}^{INV}$, and ${\eta }_{u}^{Wire}$ are the number of PV modules, installed capacity of the PV module, conversion efficiency of the PV module, inverter efficiency, and wire efficiency in the unit u and time step t, respectively. ${I}_{u,t}^{rad}$, ${I}_{u}^{nom}$, ${\beta }_{u}^{T}$, ${T}_{u,t}^{C}$, and ${T}_{u}^{Cnom}$ are the ambient solar radiation intensity, the intensity of solar radiation under standard conditions, the temperature coefficient of power of the selected PV module, the cell temperature, and the cell temperature under standard conditions of operation in the unit *u* and time step *t*., respectively.

The water withdrawal and consumption of solar-based power plants are according to Table 14.

**Table 14:** Solar PV-generated power plant's water withdrawal and consumption data^69^.

Sub-category	Water consumption (m^3^/MWh)			Water withdrawal (m^3^/MWh)		
	Median	Min	Max	Median	Min	Max
Flat paneled^a^	0.023	0.004	0.098	0.023	0.004	0.098
Concentrated PV^a^	0.11	0.09	0.30	0.11	0.09	0.30

^a^Reflecting data limitations and the nature of water use, we assume withdrawal and consumption are equal for all estimates in this category.

#### Wind energy formulation

The expected electricity generation using a wind turbine depends on the fundamentals of wind energy. The expected energy supplied by a wind turbine can be described as follows^83^:

12 $${P}_{u,t}^{WT}=\left\{\begin{array}{c}0, {u}_{u,t}<0\\ \begin{array}{c}\frac{{n}_{u}^{Wind}{\eta }_{u}^{Wind}{P}_{u}^{R\_WT}\left({u}_{u,t}^{2}{u}_{u,cu{t}_{in}}^{2}\right)}{{u}_{u,rated}^{2}-{u}_{u,cut\_in}^{2}}, {{u}_{u, cut\_in}\le u}_{u,t}\le {u}_{u, rated}\\ {n}_{u}^{Wind}{\eta }_{u}^{Wind}{P}_{u}^{R\_WT}, {{u}_{u, rated}\le u}_{u,t}\le {u}_{u, cut\_off}\\ 0, {u}_{u,t}<0\end{array}\end{array}\right.$$

where ${P}_{u,t}^{WT}$, ${P}_{u}^{R\_WT}$, ${n}_{u}^{Wind}$, and ${\eta }_{u}^{Wind}$ are the actual power generated from the wind turbine, the wind turbine rated power, the wind turbine number, the efficiency of the wind system, and the power coefficient. ${u}_{u,t}$ ,${u}_{u, cut\_in}$,${u}_{u, rated}$, and ${u}_{u, cut\_off}$ are the wind speed, the cut-in wind speed at which the turbine starts operation, the wind speed at rated power, and the cut-off wind speed, after which the wind turbine must be shut down for safety reasons.

The water withdrawal and consumption of wind power plants are based on Table 15.

**Table 15:** Wind-generated power plant's water withdrawal and consumption data^69^.

Sub-category	Water consumption (m^3^/MWh)			Water withdrawal (m^3^/MWh)		
	Median	Min	Max	Median	Min	Max
Onshore	< 0.004^a^	<< 0.004^a^	0.008	0.004	0.004	0.004
Offshore	<< 0.004^a^	<< 0.004^a^	0.004	0.008	<< 0.004^a^	0.011

^a^< 0.004 designates a value between 0.0003 and 0.002 (due to rounding), and << 1 designates a value less than 0.0003.

### Demand-side

Since the proposed framework is related to the WEF nexus, the energy demand of the water and food subsystems will be prioritized over municipal demand. Intersystem and intra-system energy demands are categorized in the framework. Intra-system energy demand includes household, transportation, commercial, construction, and mining. Intersystem energy demand is related to the energy demand of the water and food subsystems, as explained below.

#### Intersystem energy demand

##### Energy demand for water subsystem

Energy is extracted, transferred, and treated water based on water quality, source, and subsystem efficiency. Compared to surface and groundwater, seawater treatment uses more energy. In addition, the increasing demand for groundwater for irrigation has increased energy consumption and lowered groundwater levels. In the past few years, the energy required and consumed by the irrigation sector, clean water supply, and treatment has been studied^84^. Desalination and treatment plants are needed to provide clean water. Different water treatment processes use different amounts of energy. Persian Gulf countries have a large number of seawater desalination units for providing drinking water. Iran is located in a world where water resources are in crisis, so this issue is extremely important^85^. Also, salty waters are vital for water treatment in Saudi Arabia and sub-Saharan Africa^86^. This type of water requires 10–12 times more energy than other types of water^87^.

*Energy demand in the pumping sector* The energy consumption of groundwater pumping depends on the groundwater level. Generally, the higher the groundwater level, the more energy it takes to extract the water. On the other hand, surface water uses less energy than groundwater. Therefore, the pumping stations will require excess energy for water transfer if gravity does not move surface water. For example, the California State Water Project uses three percent of the state's electricity to transport 1100 km of water^88^. Equation (13) shows that the power relation required for pumping is calculated using fluid mechanics relation^89^.

13 $$Energy \, \left( {KWh} \right)\, = \;\frac{{9.8 \left( {\frac{m}{{s^{2} }}} \right) \times lift \left( m \right) \times mass \left( {Kg} \right)}}{{3.6 \times 10^{6} \times efficiency \left( \% \right)}}$$

In groundwater pumping, a subset of the water subsystem, the two components of lift and mass are known as nexus variables. These variables define the interaction between the energy and water subsystem.

*Energy for treatment of fresh raw water in treatment plants *Rivers, lakes, seas, oceans, and groundwater all have suspended solids and microorganisms that require treatment. Advanced treatment may be required to remove groundwater's insoluble ions, organic compounds, or gases. Treated water can be used in residential, commercial, industrial, and agricultural sectors^90,91^. Raw water must be treated to the standard physical and chemical quality before it is supplied to consumers. The quality of drinkable water must meet standards set by the WHO or government agencies^92^. Table 16 shows the energy consumption of different raw water treatment processes for the Iranian water treatment plant.

**Table 16:** Energy consumption intensity for treatment plant's processes^92^.

Process	Unit	Amount
Raw water pumping	KWh/m^3^	0.02–0.05
Sedimentation	KWh/m^3^	0.0005–0.001
Coagulation	KWh/m^3^	0.4–0.7
Chemical dispersion	KWh/m^3^	0.008–0.022
High-rate clarification	KWh/m^3^	0.009–0.012
Floating	KWh/m^3^	0.0095–0.0355
Gravity filters	KWh/m^3^	0.005–0.014
Surface water chlorination/de-chlorination	KWh/m^3^	0.0002–0.0005
Groundwater chlorination/de-chlorination	KWh/m^3^	0.002
The general UV irradiation process	KWh/m^3^	0.01–0.05
Ozone	KWh/m^3^	0.03–0.1
Hydranautics ultrafiltration membrane	KWh/m^3^	0.025
Microfiltration	KWh/m^3^	0.18

It should be noted that the amount of treated water is considered a nexus variable for the energy subsystem to calculate the energy consumption of this sector in the demand side of energy.

*Energy for wastewater treatment *Solid and liquid wastes contaminate the water used in the residential, commercial, and industrial sectors. For instance, water and associated gas contain pollutants and toxic substances in the oil industry. Therefore, they must be treated before reusing or discharging the produced water to water bodies. Primary, secondary, and sometimes tertiary treatments are used to remove contaminants from domestic wastewater. Table 17 shows the energy consumption for wastewater treatment in various processes for Iranian wastewater treatment plants.

**Table 17:** Energy consumption intensity for wastewater treatment plant's processes^92^.

Process	Unit	Amount
Influent wastewater pumping and collection	KWh/m^3^	0.02–0.04
Raw sewage collection and pumping	KWh/m^3^	0.04
The grit removal processes	KWh/m^3^	0.008–0.01
Aeration blowers	KWh/m^3^	0.026–0.04
Recirculation pumping in activated sludge	KWh/m^3^	0.011
Aerobic digestion	KWh/m^3^	0.5
Aeration processes	KWh/m^3^	0.13
Advanced water treatment with nitrification	KWh/m^3^	0.4–0.5
Anaerobic digestion	KWh/m^3^	0.28
Removing excess nitrates	KWh/m^3^	0.09–0.29
Dewatering stage	KWh/m^3^	0.3
Phosphorus removal	KWh/m^3^	0.8–1.6
Phosphorus removal with microfiltration membranes	KWh/m^3^	0.18

The amount of treated wastewater is considered a nexus variable for the energy subsystem to calculate the energy consumption of this sector in the demand side of energy.

##### Energy demand for food subsystem

Food is an important sector that consumes a vast amount of energy, and the price of its byproducts is inextricably linked to the cost of energy. Food byproduct prices increased substantially in 2006–2011 during the economic recession. Compared to 1990–1999, the global index of food prices increased by 104.5% from 2000 to 2012. Put another way, the energy price dramatically increased by 183.6%^93^. Thus, energy prices directly affect the food supply chain cost.

On the other hand, people in society require energy carriers to meet their demands for heat, light, traveling, Etc. Energy is used in various activities of the food subsystem, including the operation of agricultural machinery, processing, transportation, packaging, storage, and food preparation^93^. Undoubtedly, most of the energy needed in the food subsystem is consumed during the production stage. Agriculture consumes a great deal of energy, both directly and indirectly. In agriculture, direct energy consumption refers to the energy consumed by agricultural machinery, irrigation equipment, Etc. In addition, indirect energy consumption is defined as the energy required to produce fertilizers, pesticides, seeds, and insecticides. In the United States, the agricultural department has estimated that the food subsystem uses 16% of the energy budget^94^. The production stage of the food subsystem consists of the crop field, livestock, and aquaculture.

*Energy consumption in the crop fields sector *During the production stage of the food subsystem, energy is required to produce agricultural inputs such as fertilizers and pesticides to be used on the farm. The energy intensity data for producing agricultural inputs are shown in Table 18.

**Table 18:** Energy intensity data for agricultural inputs production^94^.

Agricultural inputs	Energy requirement (MJ/Kg)
Nitrogen (N)	78.1
Phosphate (P_2_O_5_)	17.4
Potassium (K_2_O)	13.7
Organic Manure	0.3
Insecticides	58
Fungicides	115
Herbicides	295
Seeds	25

The energy consumption of agricultural inputs is considered indirect energy consumption. The amount of input consumed in the agricultural field is known as the energy subsystem's nexus variable, which determines the interaction between the energy subsystem and agriculture in the energy demand side.

Also, energy carriers such as gasoline, diesel, and electricity are needed to operate agricultural machinery on agricultural land for cultivating, protecting, and harvesting. This type of energy is called direct energy consumption, which means that the energy carrier is consumed directly by machines on the farm. The direct consumed energy for agricultural machinery operation is calculated by Eq. (14)^95^:

14 $$ME=E\times \frac{G}{T}\times {Q}_{h}$$

where $ME$ is the machinery energy (MJ/ha), the production energy of the machine, which is 93.61 MJ/Kg, for the tractor, and 116 MJ/Kg for the combine, G for the weight of the machine (Kg), T for the economic life of the machine (h) and ${Q}_{h}$ is total working hours of the machine in a season. The calculated equivalent energy has been summarized in Table 19. Also, the labor energy was equaled using Eq. (15)^96^:

15 $${E}_{l}={W}_{l}\times {E}_{i}$$

where ${E}_{l}$ is the human labor energy (MJ/ha), ${W}_{l}$ is the number of workers per hectare (n/ha) and ${E}_{i}$ is the energy use per worker (MJ/n). For a man, the equivalent energy of an hour of working was supposed to be 1.96 MJ (See Table 19).

**Table 19:** Energy intensity data for agricultural activities on the farm.

Input	Unit	Energy equivalent (MJ/unit)	References
Human labor	ha	1.96	^96^
Machinery tractor	Kg	93.6	^96^
Combine	Kg	87.63	^99^
Other machinery	Kg	62.71	^99^
Diesel fuel	Lit	56.31	^100^
Water for irrigation	m^3^	0.84	^101^
Electricity	KWh	3.60	^101^
Seed (Wheat)	Kg	20.10	^101^
Seeds (bean)	Kg	14.9	^102^
Seeds (Sugar beet)	Kg	50	^103^
Seeds (Potatoes)	Kg	53	^97^
Seeds (Onion)	Kg	14.7	^104^
Seeds (Watermelon)	Kg	26.2	^105^
Seeds (Cucumber)	Kg	1	^95^
Seeds (Alfalfa)	Kg	10	^106^
Seeds (Almond)	Kg	24.08	^107^
Seeds (Walnut)	Kg	26.15	^108^
Seeds (Barley)	Kg	14.7	^101^
Seeds (Chickpea)	Kg	14.7	^96^
Seeds (Safflower)	Kg	25	^106^

The consumptions value of seed, pesticide, and fertilizer were collected through the questionnaires, and their equivalent energy per unit was then obtained, as shown in Table 19. The amount of seed energy was obtained by the following expression^97^:

16 $${E}_{s}={W}_{i}\times {E}_{i}$$

where ${E}_{s}$ is energy of seed (MJ/ha), ${W}_{i}$ is the amount of used seed (Kg/ha) and ${E}_{i}$ energy per kilogram of seed (MJ/Kg). To calculate the fuel energy, the following general equation was also applied^98^:

17 $${E}_{p}={Q}_{i}\times {E}_{i}$$

where ${E}_{p}$ is fuel energy (MJ/ha), ${Q}_{i}$ is the amount of fuel consumed (Lit/ha) and ${E}_{i}$ energy equivalent of each fuel unit (MJ/Lit). Energy equivalents of the whole inputs applied in crop production have been illustrated in Table 19.

The energy consumed by agricultural machinery in different stages of production, such as planting, planting, and harvesting, is considered as direct energy consumption. In this section, the area under cultivation of crops is considered a nexus variable for the energy subsystem. In other words, by receiving the cultivated area variable from the food subsystem, the energy subsystem can calculate the energy consumption in each simulation time step.

## Conclusion

The country of Iran is essential in terms of energy production and consumption, and the economy of Iran is mainly dependent on energy revenues. On the other hand, in the agricultural sector in Iran, energy is consumed by agricultural inputs in different parts of production, i.e., the use of machinery and agricultural inputs. Therefore, thermal and hydropower plants consume water to produce various energy carriers. Considering that Iran is suffering from water stress, the nexus of water and energy becomes very important. Also, to provide different cultivation patterns, their energy consumption should be estimated in addition to the water requirement of the crops. Therefore, to evaluate the energy system in Iran with the nexus approach, it was necessary to provide a framework.

Currently, no comprehensive framework for energy subsystems has been proposed which can be applied at a large scale and can consider the binding interactions in WEF nexus models. The supply and demand side of the energy subsystem was not captured in most of the previous WEF nexus models. Unlike the energy frameworks used within the previous WEF nexus models, which were primarily based on data, our energy framework utilized data and equations to formulate the energy subsystem's supply and demand sides. This study proposed a new energy subsystem framework based on the nexus system approach by reviewing existing literature. All the necessary data and equations must have been gathered to develop such a framework, including energy for water, food, energy for energy, and water for energy. In fact, by collecting the required data and relations, the energy subsystem was categorized into two parts: supply and demand. The energy subsystem can easily interact with the water and food subsystems in the supply and demand sectors using the information collected from the literature.

It should be noted that the supply and demand side of the energy subsystem will be given more flexibility by considering binding interactions between WEF. In addition, this framework provides a mechanism by which the water subsystem manages the allocated and consumed water, leading to a policy decision with the best outcome. Furthermore, by constituting the demand side of the energy subsystem considering the WEF nexus system approach, the food subsystem can provide optimal cropping patterns based on energy consumption within the WEF nexus system.

One of the limitations of the research was that the quality of water returned from power plants had not been evaluated due to the lack of access to data, while it is one of the critical factors in the energy subsystem that affects the quality of water resources. Also, at the watershed scale, the energy system's boundary does not match the two subsystems of water and food, and this causes inconsistency in the modeling of the nexus system. Working on modeling water withdrawal and consumption of thermal power plants with heat rate changes as research suggestions. Heat rate changes in each time step of the simulation cause the amount of water withdrawal and consumption to change and obtain a more accurate amount in the WEF nexus system.

## Data Availability

All of the required data have been presented in this article.
